# Roles of Octopamine and Dopamine Neurons for Mediating Appetitive and Aversive Signals in Pavlovian Conditioning in Crickets

**DOI:** 10.3389/fphys.2017.01027

**Published:** 2017-12-12

**Authors:** Makoto Mizunami, Yukihisa Matsumoto

**Affiliations:** ^1^Faculty of Science, Hokkaido University, Sapporo, Japan; ^2^College of Liberal Arts and Science, Tokyo Medical and Dental University, Ichikawa, Japan

**Keywords:** octopamine, dopamine, appetitive learning, aversive learning, insects, classical conditioning

## Abstract

Revealing neural systems that mediate appetite and aversive signals in associative learning is critical for understanding the brain mechanisms controlling adaptive behavior in animals. In mammals, it has been shown that some classes of dopamine neurons in the midbrain mediate prediction error signals that govern the learning process, whereas other classes of dopamine neurons control execution of learned actions. In this review, based on the results of our studies on Pavlovian conditioning in the cricket *Gryllus bimaculatus* and by referring to the findings in honey bees and fruit-flies, we argue that comparable aminergic systems exist in the insect brain. We found that administrations of octopamine (the invertebrate counterpart of noradrenaline) and dopamine receptor antagonists impair conditioning to associate an olfactory or visual conditioned stimulus (CS) with water or sodium chloride solution (appetitive or aversive unconditioned stimulus, US), respectively, suggesting that specific octopamine and dopamine neurons mediate appetitive and aversive signals, respectively, in conditioning in crickets. These findings differ from findings in fruit-flies. In fruit-flies, appetitive and aversive signals are mediated by different dopamine neuron subsets, suggesting diversity in neurotransmitters mediating appetitive signals in insects. We also found evidences of “blocking” and “auto-blocking” phenomena, which suggested that the prediction error, the discrepancy between actual US and predicted US, governs the conditioning in crickets and that octopamine neurons mediate prediction error signals for appetitive US. Our studies also showed that activations of octopamine and dopamine neurons are needed for the execution of an appetitive conditioned response (CR) and an aversive CR, respectively, and we, thus, proposed that these neurons mediate US prediction signals that drive appetitive and aversive CRs. Our findings suggest that the basic principles of functioning of aminergic systems in associative learning, i.e., to transmit prediction error signals for conditioning and to convey US prediction signals for execution of CR, are conserved among insects and mammals, on account of the fact that the organization of the insect brain is much simpler than that of the mammalian brain. Further investigation of aminergic systems that govern associative learning in insects should lead to a better understanding of commonalities and diversities of computational rules underlying associative learning in animals.

## Introduction

Elucidation of neural systems that mediate appetite and aversive signals in associative learning is an important subject in neuroscience. By associative learning, animals can acquire knowledge in their environments, which allow them, for example, to find suitable food, avoid toxic compounds, and escape from predators. Efforts have been made to elucidate neural systems mediating appetitive and aversive signals in associative learning in many animals, including mammals (Schultz, 2013, 2015), insects (Hammer and Menzel, 1998; Schwaerzel et al., 2003; Mizunami and Matsumoto, 2010; Waddell, 2013), and mollusks (Hawkins and Byrne, 2015). Prediction error, i.e., the discrepancy, or error, between the actual unconditioned stimulus (US) and the predicted US, represents a key determinant for whether a US-paired stimulus is learned (Rescorla and Wagner, 1972; Schultz, 2013, 2015). There is evidence that some classes of midbrain dopamine neurons mediate prediction error signals for appetitive events (Schultz, 2013, 2015), and some researchers have suggested that other classes of midbrain dopamine neurons mediate prediction error signals for aversive events (Matsumoto and Hikosaka, 2009; Matsumoto H. et al., 2016). Other classes of midbrain dopamine neurons control the execution of both appetitively and aversively learned actions (Berridge et al., 2009; Bromberg-Martin et al., 2010).

This review deals with results of our studies on the roles of biogenic amines in appetitive and aversive learning in crickets. Crickets are useful insects for the study of neurotransmitter mechanisms of learning and memory. First, they have excellent capabilities of olfactory and visual learning. For example, they exhibit lifetime olfactory memory (Matsumoto and Mizunami, 2002a), simultaneous memorization of seven pairs of odors (Matsumoto and Mizunami, 2006), context-dependent discriminatory learning (Matsumoto and Mizunami, 2004), and higher-order associative learning such as second-order conditioning (Mizunami et al., 2009) and sensory preconditioning (Matsumoto et al., 2013a). They also exhibit excellent capability to learn color and pattern of visual targets (Unoki et al., 2006; Nakatani et al., 2009; Matsumoto et al., 2013b). Second, applications of pharmacological studies (Unoki et al., 2005, 2006; Matsumoto et al., 2006, 2016; Matsumoto Y. et al., 2009; Mizunami et al., 2014; Sugimachi et al., 2016), gene knockdown by RNA interference (RNAi; Takahashi et al., 2009; Awata et al., 2016), and genome editing by the CRISPR/Cas9 system (Awata et al., 2015) are feasible, thereby greatly facilitating the analysis of molecular basis of learning and memory. Indeed, it can be stated that crickets are one of the best insect models for pharmacological analysis of learning and memory (Mizunami et al., 2013). Third, much information on the brain and behavior of crickets has been obtained as crickets have been used in diverse neuroethological studies (Stevenson and Schildberger, 2013; Hedwig, 2016). We first deal with the recent debate about whether appetite and aversive signals are conveyed by octopamine and dopamine neurons, respectively, as has been suggested in honey bees and crickets, or whether both appetitive and aversive signals are mediated by dopamine neurons, as has been suggested in fruit-flies. Next, we discuss the results of our studies suggesting (1) that activations of octopamine neurons and activation of dopamine neurons are needed for responding to an appetitive conditioned stimulus (CS) and an aversive CS, respectively, and (2) that conditioning is governed by US prediction error and that octopamine neurons mediate the prediction error signals for appetitive learning.

## Conditioning procedures

We have established four different conditioning procedures for crickets (Matsumoto and Mizunami, 2000, 2002a,b; Matsumoto et al., 2015). Among them, we used a “classical conditioning and operant testing procedure” (Matsumoto and Mizunami, 2002b; Matsumoto et al., 2003), which is based on the transfer of memory formed during classical conditioning to an operant testing situation. Crickets were individually placed in a beaker and deprived of drinking water for 3 days to enhance motivation to uptake water. For conditioning of an odor (CS) with water US, a filter paper soaked with an odor was presented to the antennae of the cricket for 3 s, and then a drop of water was applied to the mouth. For conditioning of an odor with sodium chloride US, an odor was presented to the antennae and then a drop of 20% sodium chloride solution was applied to the mouth. Crickets were eager to drink water when it was applied to the mouth, whereas they immediately retracted from sodium chloride solution, indicating that the former serves as an appetitive stimulus and that the latter serves as an aversive stimulus. Odor preferences of individual crickets were tested before and after conditioning. In the test, crickets were individually placed in a test chamber and allowed to freely visit two odor sources, a conditioned odor and a control odor, for 4 min. The time that the cricket spent exploring each odor source with its mouth or palpi was recorded for evaluation of the relative odor preference of each cricket.

For conditioning of a visual pattern, presentation of water or sodium chloride solution to the mouth was paired with either a black-center and white-surround pattern or with its reverse pattern (Unoki et al., 2006). In the pattern preference test, the two patterns were simultaneously presented on the wall of the test chamber, and the time that the cricket spent touching each of the patterns was recorded for evaluating the relative preference between the two patterns. For color conditioning, crickets were presented with purple and green disks paired with water or sodium chloride US (Nakatani et al., 2009), and the two disks were presented simultaneously on the wall of the test chamber for the color preference test.

We also used conditioning of maxillary palpi extension response (MER) with odor CS and water or sodium chloride US, which allowed us to investigate the memory acquisition process (Matsumoto et al., 2015). Crickets often extend their maxillary palpi and then vigorously swing them when a drop of water is applied to their antenna or to the mouth, and we refer to this behavior as the MER. Crickets often exhibited MER to some odors such as vanilla and maple odors, whereas they rarely exhibited MER to other odors such as peppermint and apple odors. We showed that the MER to peppermint or apple odor is increased by pairing the odor with water US (Matsumoto et al., 2015). MER conditioning is analogous to the conditioning of proboscis extension responses (PERs) with odor CS and sucrose US in honey bees (Menzel and Giurfa, 2006; Giurfa and Sandoz, 2012). Moreover, we also observed that the MER to vanilla or maple odor is decreased by pairing an odor with sodium chloride. Therefore, MER conditioning allows appetitive conditioning and aversive conditioning to be achieved in a similar experimental situation, as in the case of a classical conditioning and operant testing procedure.

## Roles of octopamine and dopamine in appetitive and aversive learning

Previous studies done in the honey bee *Apis mellifera* (Hammer and Menzel, 1998) and the fruit-fly *Drosophila melanogaster* (Schwaerzel et al., 2003) suggested that octopamine and dopamine neurons play critical roles in appetitive and aversive olfactory conditioning, respectively (for alternative view, see later section). We first investigated whether this was the case in crickets using a classical conditioning and operant testing procedure (Unoki et al., 2005). Crickets were injected with an octopamine receptor antagonist (epinastine or mianserin) into the hemolymph prior to the conditioning of an odor with water. In a post-training retention test, they did not exhibit an increase of preference for the odor conditioned with water. However, crickets showed normal scores of aversive conditioning with sodium chloride, and the scores being as high as those for control crickets that had been injected with cricket's saline solution. The latter observation indicates that octopamine receptor antagonists do not impair sensory function, motor function, or motivation necessary for learning. We also observed that crickets injected with a dopamine receptor antagonist (fluphenazine, chlorpromazine, spiperone, or flupentixol: Different dopamine receptor types are not discriminated by these drugs, see Mustard et al., 2005) exhibited no aversive learning with sodium chloride US, but appetitive learning with water US was unaffected. Sensory function, motor function, or motivation necessary for learning is not affected by dopamine receptor antagonists. Similar results were obtained in a recent study using olfactory conditioning of MER (Matsumoto et al., 2015). We, thus, suggest that octopamine codes for appetitive signals, and that dopamine neurons transmit aversive signals in two different forms of Pavlovian conditioning in crickets. Notably, crickets that were injected with octopamine or dopamine receptor antagonist exhibited a normal appetitive or aversive response, respectively, when water or sodium chloride solution was applied to the mouth. Hence, these neurotransmitters are not involved in the execution of a behavioral response to appetitive or aversive US.

We also investigated whether the blockade of octopaminergic and dopaminergic transmissions impairs appetitive and aversive conditioning, respectively, of a visual pattern (Unoki et al., 2006) and a color cue (Nakatani et al., 2009). For conditioning of a visual pattern, we observed that crickets injected with an octopamine receptor antagonist (epinastine or mianserin) exhibited no appetitive learning with water, but they exhibited normal aversive learning with sodium chloride solution. In contrast, crickets injected with a dopamine receptor antagonist (spiperone, chlorpromazine, or fluphenazine) exhibited no aversive learning, but appetitive learning was unaffected (Unoki et al., 2006). In color conditioning, crickets injected with an octopamine receptor antagonist (epinastine or mianserin) exhibited impaired appetitive color learning, but aversive color learning was unaffected. In contrast, crickets injected with a dopamine receptor antagonist (flupentixol, fluphenazine, or chlorpromazine) exhibited impaired aversive color learning, whereas appetitive color learning was unaffected (Nakatani et al., 2009). The results indicate that octopamine and dopamine neurons convey signals about an appetitive vs. an aversive US, regardless of the specific paradigm used, thereby suggesting the action of separate neurotransmitter systems to mediate appetitive and aversive signals, respectively, in associative learning in crickets.

## Roles of octopamine and dopamine in appetite and aversive learning confirmed by RNAi and transgenic crickets

Recent studies on neurotransmitters mediating appetitive and aversive signals for Pavlovian conditioning in the fruit-fly, have yielded conclusions that differ from those obtained in crickets (Burke et al., 2012; Liu et al., 2012). In the fruit-fly, different sets of dopamine neurons mediate appetitive and aversive signals, such as sucrose and electric shock signals, respectively, to intrinsic neurons (Kenyon cells) of the mushroom body (MB), via the type 1 dopamine receptor Dop1, in the MB lobes (Kim et al., 2007; Burke et al., 2012; Liu et al., 2012; Perry and Barron, 2013; Waddell, 2013; Ichinose et al., 2015). Octopamine neurons in the subesophageal ganglion receive sweet taste signals from sugar receptor neurons and relay the signals to dopamine neurons in the protocerebrum that project to the MB lobes (Burke et al., 2012). Therefore, octopamine neurons have a peripheral role for relaying sweet taste signals (Huetteroth et al., 2015), whereas dopamine neurons transmit appetitive US signals to the MB to associate them with an olfactory CS (Burke et al., 2012). Considering that octopamine neurons play roles in mediating appetitive signals in flies, a critical difference between flies and crickets is that dopamine neurons mediate appetitive signals in flies but not in crickets. We considered three possible reasons for this difference, and we investigated them in crickets. The first possible reason is the use of different methods to inhibit dopaminergic signaling: while the use of transgenic techniques in flies allows a sophisticated way to silence dopamine or octopamine signaling, efficacies and specificities of antagonists used in the cricket may not be perfect. For example, a recent study in honey bees suggested that epinastine and mianserin antagonize not only OA1 octopamine receptors but also Dop2 dopamine receptors (Beggs et al., 2011), which raises the possibility that impairment of appetitive learning by epinastine and mianserin might be mediated via blockade of Dop2 receptors, instead of or in addition to OA1 receptors. The second possible reason is the use of different kinds of appetitive US for conditioning. We used water as US in our studies on crickets, whereas sucrose was used in studies on flies except for two studies using water (Lin et al., 2014; Shyu et al., 2017). We, thus, considered the possibility that dopamine conveys sucrose US but not water US in crickets. The third possible reason is that neurotransmitters mediating appetitive signals are not the same in flies and crickets.

For clarifying the issues discussed above, we prepared transgenic crickets with *Dop1* gene knockout using the CRISPR/Cas9 system [clustered regularly interspaced short palindromic repeats (CRISPR)/CRISPR-associated protein 9 (Cas9) system; Awata et al., 2015]. Dop1 is known to be highly enriched in the MB in fruit-flies (Kim et al., 2007) and honey bees (Mustard et al., 2005). *Dop1* knockout crickets exhibited no obvious abnormality in behavior and external morphology. Our conditioning experiments showed that *Dop1* knockout crickets exhibited no aversive learning with sodium chloride US but exhibited normal appetitive learning with water US or sucrose US (Awata et al., 2015). The latter finding indicates that the impairment of aversive learning was not due to the impairment of sensory or motor functions or motivation necessary for learning and for responding to the conditioned odor in the post-training test. The results suggest that Dop1 participates in aversive learning with sodium chloride but not in appetitive learning with water or sucrose in crickets. This differ from the findings in flies in which Dop1 is required for both appetitive learning with water or sugar US and aversive learning with electric shock (Kim et al., 2007; Burke et al., 2012; Liu et al., 2012).

It could be argued, however, that knockout of *Dop1* might have caused an abnormality in the development of neural circuits in the brain necessary for aversive learning, not that Dop1 has acute roles in learning in adults. For further clarification of this issue, we investigated the effects of silencing the expression of genes that code the OA1 octopamine receptor and the Dop1 and Dop2 dopamine receptors by RNAi in adult crickets (Awata et al., 2016). In those studies, we used olfactory conditioning of MER to investigate the effect of gene silencing on the acquisition process. Crickets were injected with dsRNA-targeting *OA1, Dop1*, or *Dop2* into the hemolymph and subjected 2 days later to conditioning trials to associate an odor with water or sodium chloride. Studies with quantitative real-time PCR (qPCR) confirmed a significant reduction in the mRNA level of each gene 2 days after dsRNA injection. *OA1*-silenced crickets exhibited no appetitive learning, but they exhibited normal scores in aversive learning. In contrast, *Dop1*-silenced crickets exhibited no aversive learning but exhibited normal scores in appetitive learning. *Dop2*-silenced crickets, as well as control crickets injected with *DsRed* dsRNA, showed normal scores in both appetitive learning and aversive learning. We, thus, conclude that octopamine mediates appetitive signals via OA1 receptors, whereas dopamine mediates aversive signals via Dop1 receptors in crickets. The perfect agreements of the results of pharmacological, transgenic, and RNAi studies provide decisive evidence that neurotransmitters and receptors that mediate appetitive signals indeed differ in crickets and flies. Our findings in crickets are in accordance with the findings in honey bees, where it has been suggested that appetitive learning is mediated by octopamine neurons via OA1 receptors (Hammer, 1993; Hammer and Menzel, 1998; Farooqui et al., 2003) and that aversive learning is mediated by dopamine neurons (Vergoz et al., 2007; the types of dopamine receptors involved are not known). Neurotransmitters involved in appetitive and aversive learning in other species of insects, however, remain elusive. More studies on various species of insects are needed to elucidate the diversity and evolutionary history of the neurotransmitters in mediating appetite and aversive signals in insects.

In associative learning in mammals, there is evidence that some classes of midbrain dopamine neurons convey signals about appetitive events (Schultz, 2013, 2015), whereas other classes may convey signals about aversive events (Matsumoto and Hikosaka, 2009; Matsumoto H. et al., 2016) (for more details, see Discussion in a later section). Hence, biogenic amines mediating appetitive signals are not the same between crickets and mammals, although the roles of dopamine in mediating aversive signals may be conserved between them. Dopamine has been reported to mediate appetitive signals in the mollusk *Aplysia* (Brembs et al., 2002). The origin of octopamine signaling for mediating appetite signals in crickets remains to be studied.

## Roles of octopamine and dopamine in execution of appetitive and aversive conditioned responses

We next investigated whether administration of octopamine and dopamine receptor antagonists affects the performance of conditioned responses (CRs; or memory retrieval) after appetitive or aversive conditioning. Crickets were subjected to appetitive or aversive olfactory conditioning and then they received an injection of either octopamine or dopamine receptor antagonist before a retention test (Mizunami et al., 2009). Crickets injected with an octopamine receptor antagonist (epinastine) exhibited no CR to the odor associated with water, whereas they exhibited normal CR to the odor associated with sodium chloride. The latter indicates that epinastine had no effect on sensory and motor functions as well as the motivation necessary to perform a CR. This is in contrast to the finding that crickets injected with a dopamine receptor antagonist (flupentixol) exhibited no CR to the odor conditioned with aversive US but that they showed a normal CR to the odor conditioned with appetitive US. The latter finding indicates that flupentixol had no effect on sensory and motor functions as well as the motivation necessary to perform a CR. After recovery from the effect of the antagonists, crickets exhibited normal CRs. These observations are in accordance with the evidence from honey bees in which a disruption of antennal lobe (i.e., the primary olfactory center) octopaminergic transmission by either the octopamine receptor antagonist mianserin or RNAi of the *OA1* gene, disrupted the execution of an appetitive CR (or of appetitive memory retrieval; Farooqui et al., 2003). Moreover, visual pattern conditioning for appetitive or aversive CRs was impaired by injections of an octopamine or dopamine receptor antagonist, respectively (Mizunami et al., 2009). Therefore, we conclude that the execution of appetitive and aversive CRs for olfactory and visual cues requires intact octopaminergic or dopaminergic transmission, respectively.

**Figure 1:**
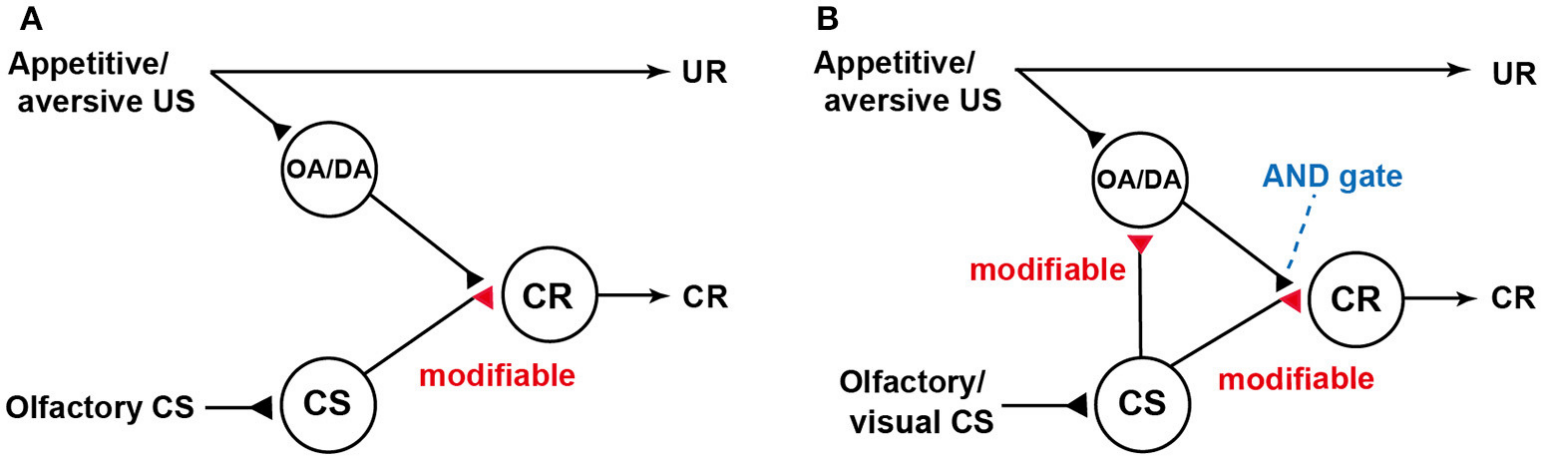
Models of classical conditioning in flies and crickets. **(A)** A model proposed to account for the roles of intrinsic and extrinsic (output) neurons of the mushroom body (MB) in olfactory conditioning in fruit-flies (Schwaerzel et al., 2003). Octopamine neurons and dopamine neurons (“OA/DA” neurons) convey signals for appetitive and aversive US, respectively (In recent models in fruit-flies, “OA/DA” neurons have been replaced to different sets of DA neurons,). “CS” neurons, which convey signals for olfactory CS, make synaptic connections with “CR” neurons that induce a CR, which mimics an unconditioned response (UR). “OA/DA” neurons make synaptic connections with axon terminals of the “CS” neurons. The efficacy of the “CS-CR” synaptic connection is strengthened by coincident activation of “CS” neurons and “OA/DA” neurons by conditioning. For recent elaborations of the model in fruit-flies, see Hige (2017). **(B)** Our model of classical conditioning proposed for crickets (Mizunami et al., 2009). The model assumes that (1) efficacy of synaptic transmission from “CS” neurons to “OA/DA” neurons is strengthened by conditioning and that (2) coincident activation of “OA/DA” neurons and “CS” neurons is needed to activate “CR” neurons (AND gate) and to produce a CR. Synapses for which the efficacies are modifiable by CS-US pairings are shown as open triangles and marked “modifiable”. Following the terminology of learning theories in mammals, the model in flies is characterized as an S-R model assuming formation of CS-CR connections, while our model is characterized as an S-R and S-S hybrid model assuming formation of CS-CR and CS-US connections. Modified from Mizunami et al. (2009).

Our findings were not in accordance with a neural model of classical conditioning proposed by Schwaerzel et al. (2003) (Figure 1A), which was designed to account for the roles of intrinsic neurons (Kenyon cells) and extrinsic (output) neurons of the MB lobes in conditioning of an odor with sugar or electric shock US in the fruit-fly. The model assumed that (1) “CS” neurons (Kenyon cells) carry CS signals and make synaptic connections with dendrites of “CR” neurons (output neurons of the lobes), activation of which leads to a CR, (2) these synaptic connections are silent or very weak prior to conditioning, (3) octopamine and dopamine neurons projecting to the lobes (“OA/DA” neurons) convey signals for appetitive and aversive US, respectively, and make synaptic connections with axon terminals of “CS” neurons (in recent models of fruit-flies, “OA/DA” neurons have been replaced with different sets of DA neurons. See Burke et al., 2012; Liu et al., 2012.), and (4) coincident activation of “CS” neurons and “OA/DA” neurons in conditioning strengthens the efficacy of synaptic transmission from “CS” neurons to “CR” neurons.

We proposed a novel neural model of classical conditioning for the cricket (Mizunami et al., 2009) with minimal modifications of the model proposed for the fruit-fly by Schwaerzel et al. (2003). In our model (Figure 1B), it is assumed that (1) coincident activation of “CS” neurons and “OA/DA” neurons is required for activating “CR” neurons (AND gate) and producing a CR after conditioning and (2) simultaneous activation of “CS” and “OA/DA” neurons from CS/US pairing strengthens the synaptic connection between “CS” and “OA/DA” neurons. Following conventional learning theory, the model proposed for the fly is termed as S-R (or CS-CR) model, as it assumes the formation of stimulus-response (CS-CR) sensorimotor pathways by conditioning, whereas our model is termed as S-R and S-S (or CS-US) hybrid model, which assumes the formation of S-R connections and CS-US connections; the latter of which enables the CS to activate internal representation of US (for details, see Mizunami et al., 2009; Mizunami and Matsumoto, 2010). In our model, the extent by which the CS activates “OA/DA” neurons represents the extent by which the CS predicts the US, and the requirement of activated “OA/DA” neurons for execution of a CR indicates that US prediction guides the execution of the CR, as assumed in S-S learning theory (see Mizunami et al., 2009; Mizunami and Matsumoto, 2010). This is analogous to the findings that some classes of midbrain dopamine neurons govern the execution of learned actions in Pavlovian conditioning in mammals (Balleine et al., 2007; Bromberg-Martin et al., 2010). Following the terminology of human psychology, it has been stated that dopamine neurons confer a “wanting” attribute to the CS to drive actions to seek a US (Berridge et al., 2009). Our findings suggest that motivational mechanisms that govern the execution of a CR in insects are analogous to those in mammals.

## Roles of octopamine in mediating prediction error for appetite US

Finally, we address the question of what computational rules govern the learning process in crickets. In mammals, a discrepancy, or an error, between the actual US and the predicted US facilitates the classical conditioning for a stimulus paired with the US (Schultz, 2013, 2015). This theory emerged from the finding of “blocking” in rats (Kamin, 1969), in which pairing of stimulus X with US, and subsequent pairing of a compound of stimulus X and another stimulus Y with the US, blocked the learning of stimulus Y. Kamin (1969) argued that blocking requires surprise for learning, whereas learning does not occur when the animal fully predicts the occurrence of the US, and this argument was formulated into the prediction error theory of the Rescorla–Wagner model (Rescorla and Wagner, 1972). Activation of dopamine neurons in the mammalian ventral tegmental area is thought to mediate the prediction error signals for rewarding events in Pavlovian and instrumental conditioning (Waelti et al., 2001; Schultz, 2013, 2015). However, blocking can also be accounted for theories other than the prediction error theory, such as attentional theory (Mackintosh, 1975; Pearce and Hall, 1980) and retrieval theory (Miller and Matzel, 1988), and decisive evidence to discriminate prediction error theory from competitive theories has not been obtained in any learning systems of animals (Miller et al., 1995; Pearce, 2008; Mazur, 2013). Therefore, unambiguous demonstration of the validity of the prediction error theory remains to be achieved.

We performed experiments to investigate whether blocking occurs in classical conditioning in crickets (Terao et al., 2015). No convincing evidence of blocking has yet been obtained in any species of insects. In honey bees, for example, it has been concluded that blocking is not a robust phenomenon (Guerrieri et al., 2005; Blaser et al., 2006, 2008). We first investigated whether blocking of learning of an odor occurs. One group of crickets (blocking group) was subjected to pairing of a visual pattern (X) with a water US (reward) (X+ training) and then subjected to pairing of a pattern (X)-odor (Y) compound with water (XY+ training). An unpaired group received unpaired presentations of a visual pattern (X) and reward and then XY+ training. The blocking group exhibited no learning of the odor (Y), whereas the unpaired group exhibited normal learning of the odor (Y). We found that blocking of visual pattern learning also occurs (Terao et al., 2015). In a test of the prediction error theory, 1-trial XY+ conditioning should be successful, whereas in attentional theory, it should not be successful. We observed successful 1-trial XY+ conditioning, which matches with the prediction error theory but not with the attentional theory (Terao et al., 2015).

**Figure 2:**
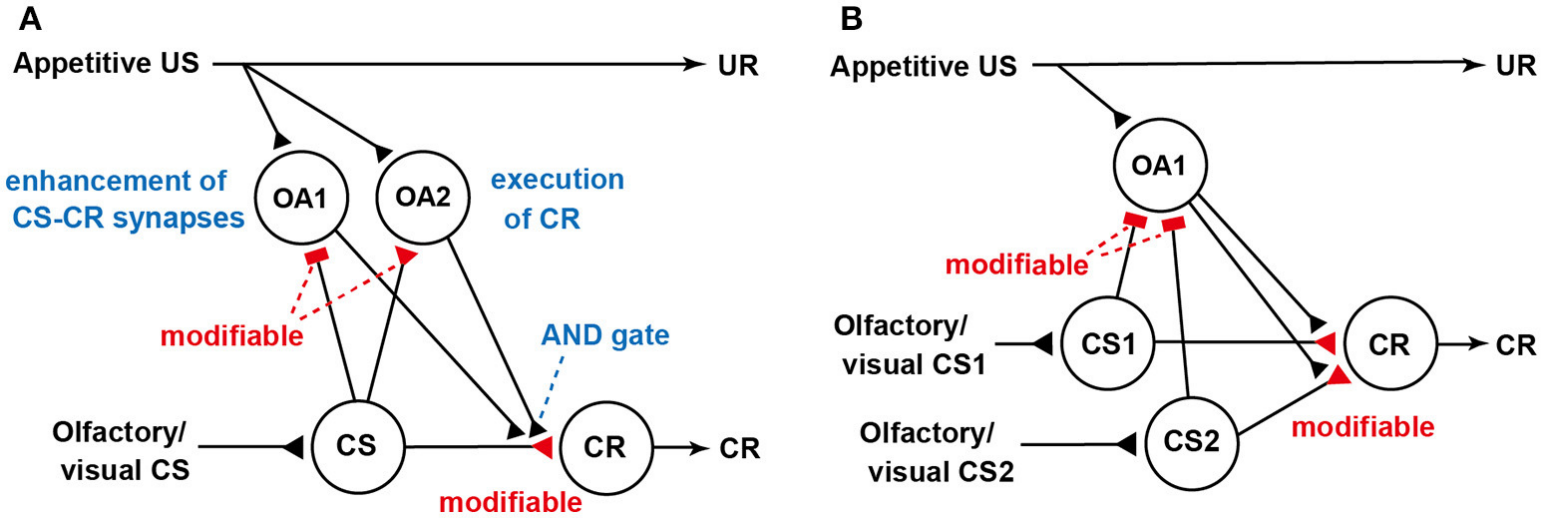
Our updated model of the roles of octopamine neurons in appetitive conditioning. **(A)** Our updated model of classical conditioning established by modifying our previous model (Figure 1B) to match the prediction error theory (Terao et al., 2015). The model retains basic feature of the S-R and S-S hybrid model but assumes the presence of two classes of octopamine neurons, namely, “OA1” neurons that govern enhancement of “CS-CR” synapses (but not execution of a CR) and “OA2” neurons that govern execution of a CR or memory retrieval (but not a conditioning process). OA2 neurons, but not OA1 neurons, govern the “AND gate”. In this figure, we focus on the roles of “OA1” neurons: “OA2” neurons are not illustrated for simplicity. We assume that “OA1” neurons receive no or very weak inhibitory synaptic input from “CS” neurons before training and that the efficacy of the inhibitory synapses is strengthened by CS-US pairing in training. During training, “OA1” neurons receive excitatory synaptic input (triangle) representing actual US and inhibitory input (rectangle) from “CS” neurons representing “predicted US” from the CS. Thus, activities of “OA1” neurons represent US prediction errors. Synapses for which the efficacies are modifiable by CS-US pairings are shown as open rectangles or open triangles and are marked “modifiable”. **(B)** The model accounts for blocking and auto-blocking. In the figure, “OA2” neurons are omitted to focus on the roles of “OA1” neurons. The model assumes that pairing of a stimulus (CS1) with appetitive US leads to (1) enhancement of inhibitory pathways from “CS1” neurons to “OA1” neurons and (2) enhancement of excitatory synapses from “CS1” neurons to “CR” neurons. During pairing of a compound of CS1 and CS2 with US after sufficient repetition of CS1-US pairing trials, “OA1” neurons are inhibited by activation of “CS1” neurons and thus activation of “OA1” neurons in response to US presentation is inhibited. As a result, enhancement of “CS2-OA1” synapses and “CS2-CR”synapses, in which “CS2” neurons mediate CS2, does not occur. Therefore, no learning of CS2 occurs. The model also predicts that injection of an octopamine receptor antagonist before CS1-US conditioning trials impairs enhancement of “CS1-CR” synapses but not “CS-OA1” synapses. Therefore, no enhancement of “CS1-CR” synapses should occur in subsequent training even after recovery from the effect of the drug. We refer to this phenomenon as auto-blocking. Modified from Terao et al. (2015).

We revised our previous model (Figure 1B; Terao et al., 2015) for Pavlovian conditioning, thereby matching the prediction error theory (Figure 2A). How this model accounts for blocking is shown in Figure 2B. We noticed that the model predicted that the application of an octopamine receptor antagonist (epinastine) before Y+ training impairs the learning of Y but does not disrupt the formation of reward prediction by Y (see legend of Figure 2). Therefore, the model predicts that crickets that received Y+ training under the condition of application of epinastine and then Y+ training after recovery from the effect of epinastine exhibit no learning of Y. Indeed, crickets that received such training exhibited no learning of Y (Terao et al., 2015). The “auto-blocking” phenomenon can be easily accounted for by the prediction error theory. However, it cannot be accounted for by any of the competitive theories, as these theories assume cue competition to account for blocking, but it does not occur in an auto-blocking experiment (Terao et al., 2015). The occurrence of blocking and auto-blocking in the same learning system of the same species provides rigorous evidence for validity of the prediction error theory. Moreover, our observation that injection of an octopamine receptor antagonist leads to auto-blocking suggests that reward prediction error signals in crickets are mediated by octopamine neurons. Further neuroanatomical and electrophysiological studies of dopamine neurons are needed to elucidate neural circuit mechanisms for computation of the prediction error in crickets. Investigation is also needed to determine whether dopamine neurons mediate prediction error for aversive US.

## Conclusions

Most animals possess neural mechanisms that allow modification of their behavior for receiving appetitive stimuli and avoiding aversive stimuli. We showed that some octopamine and dopamine neurons play critical roles in appetitive and aversive learning, respectively, and more specifically, the octopamine neurons mediate reward prediction error in appetitive learning in crickets. Moreover, we suggested that some octopamine and dopamine neurons mediate signals about the extent by which the CS predicts the US and such signals drive appetitive and aversive CRs, respectively. Those roles of aminergic neurons in crickets match the S-S learning theory (see Mizunami et al., 2009) and are analogous to the roles of midbrain dopaminergic neurons in the execution of learned actions in mammals (Balleine et al., 2007; Berridge et al., 2009; Bromberg-Martin et al., 2010). We propose that the basic principles of information processing in associative learning are conserved among insects and mammals, on account of the fact that the organization of the insect brain is much simpler than that of the mammalian brain (Mizunami et al., 1999, 2004; Menzel and Giurfa, 2006, Menzel, 2012). Further studies on insect Pavlovian conditioning should pave the way for elucidating the diversity and evolution of associative learning mechanisms in animals.

In addition, our studies have demonstrated that crickets are one of most suitable animals for pharmacological analysis of learning and memory, and crickets may, thus, also be efficient model animals for screening drugs that affect motivational states of animals, and such screening may contribute to therapeutic applications in humans.

## Author contributions

MM and YM wrote the manuscript and approved the final version.

### Conflict of interest statement

The authors declare that the research was conducted in the absence of any commercial or financial relationships that could be construed as a potential conflict of interest.
